# 

**Published:** 1859-10

**Authors:** 


### CONTENTS.

Professional Intercourse........ 153
Dental Quackery........ 160
Random Thoughts. By GEO. WATT........ 172
National Society........ 175
Soldering made Easy........ 181
Dental Fees, By B. WOOD, M. D., Dentist,........ 183
N. B. Slayton........ 189
Remarks of Prof. McQuillen on Nitric Acid Treatment of Exposed Pulps. By J. TAFT,. 194
Editorial,........ 197
Superior Stone........ 198
A Question—Protrusion........ 198
Ohio College of Dental Surgery........ 199
Crystal Gold........ 199
A New Work........ 200

